# Swedish norms for the Strengths and Difficulties Questionnaire for children 3–5 years rated by parents and preschool teachers

**DOI:** 10.1111/sjop.12606

**Published:** 2019-12-12

**Authors:** Anton Dahlberg, Elisabet Fält, Ata Ghaderi, Anna Sarkadi, Raziye Salari

**Affiliations:** ^1^ Child Health and Parenting (CHAP) Department of Public Health and Caring Sciences Uppsala University Sweden; ^2^ Division of Psychology Department of Clinical Neuroscience Karolinska Institutet Sweden

**Keywords:** Strengths and difficulties questionnaire, psychometrics, Sweden, norms, preschool children, mental health

## Abstract

The Strengths and Difficulties Questionnaire (SDQ) is a widespread tool for assessing behavior problems in children and adolescents. Despite being investigated thoroughly concerning both validity and reliability, peer reviewed studies that provide norms, especially for preschool children, are lacking. This paper provides Swedish norms using data from a large community sample of children aged 3–5, based on mothers’, fathers’, and preschool teacher’s ratings. Preschool teachers’ ratings were generally lower than parents’ ratings, which contradicts some previous studies. Differences between girls and boys were found, suggesting that boys display higher levels of behavior problems. Lower parental education and country of origin outside of Sweden were also associated with more difficulties. Cut‐offs are presented for each age group, gender and rater category. Population‐specific norms and percentile cut‐offs provided in this study facilitate identifying children in need of interventions in paediatric care and enable cross‐country comparisons of children’s mental health problems.

INTRODUCTION

Mental health problems in children are common worldwide (Kieling, Baker‐Henningham, Belfer *et al.*, 2^2011^) and delivering early interventions is crucial, as some emotional and behavioral problems developed in childhood show stability over time (Caspi, Moffitt, Newman & Silva, 1996) and can increase in severity or become persistent (Fergusson, Horwood & Ridder, 2005; Hofstra, van der Ende & Verhulst, 2002). Recognizing young children in need of support can thus have immediate effects on the child’s wellbeing and benefit the child’s health in a long‐term perspective.

Early identification and treatment of behavioral problems in childhood calls for valid and reliable screening instruments. The Strengths and Difficulties Questionnaire (SDQ) is a brief questionnaire for identifying emotional and behavioral problems among children and adolescents (Goodman, 1997). In this study, we report Swedish norms for the parent and preschool teacher version of the SDQ for young children.

The SDQ was developed by Goodman in the 1990s (Goodman, 1997) as an extension of the established Rutter questionnaires (Elander & Rutter, 1996). In order to increase parental compliance, Goodman designed the SDQ to focus on strengths as well as difficulties (Goodman, 1994). The combination of positive and negative statements regarding psychological attributes and behaviors are considered to increase acceptability of the instrument, and to make it suitable for use in community samples. The SDQ is available for 2–17‐year‐olds in both parent and teacher versions (as well as a self‐rated version from 11 years). It displays adequate psychometric qualities overall (Goodman, 2001; Stone, Otten, Engels, Vermulst & Janssens, 2010), including acceptable construct validity when used on preschool children (Croft, Stride, Maughan & Rowe, 2015; Dahlberg, Ghaderi, Sarkadi & Salari, 2018). While being a relatively short questionnaire, the SDQ is still comparable to the similar but lengthier Child Behavior Checklist (CBCL), displaying moderate to high correlations on total and equivalent subgroup scores (Goodman & Scott, 1999; Klasen, Woerner, Wolke *et al.*, 2000). In addition, the SDQ was designed to meet the needs of both clinicians and researchers and can be used for screening purposes (Goodman, Ford, Simmons, Gatward & Meltzer, 2000), as part of a clinical assessment (Goodman, Renfrew & Mullick, 2000) or as a research tool. Sensitivity studies have found that the SDQ identifies 70–90% of children with conduct, hyperactivity, depressive, and some anxiety disorders (Goodman, Ford, *et al.*, 2000).

The SDQ consists of 25 items classified into five subscales, four of which refer to difficulties: emotional symptoms, conduct problems, hyperactivity/inattention, and peer problems; and one subscale that measures strengths namely prosocial behavior (Goodman, 2001). Summing up the scores from the difficulties subscales generates a total difficulties score. Higher total difficulties scores indicate more difficulties. The SDQ is also available in versions with an impact supplement (Goodman, 1999), which enables the respondent to report on perceived burden and distress.

Although the SDQ is commonly used as a screening and research tool in different countries and exists in numerous language versions, normative data are only available from a limited number of countries, ages and informants (see http://www.sdqinfo.org). Previous studies on the psychometric properties of the SDQ have shown that norms vary across cultural settings (Aiko & Yoko, 2014; Borg, Kaukonen, Joukamaa & Tamminen, 2014; Bourdon, Goodman, Rae, Simpson & Koretz, 2005; Kremer *et al.*, 2015; Lai *et al.*, 2010; Maurice‐Stam *et al.*, 2018; Niclasen, Teasdale, Andersen, Skovgaard, Elberling & Obel, 2012; Tobia & Marzocchi, 2018; Woerner, Becker & Rothenberger, 2004). Hence, to use the SDQ in research for cross‐country comparisons of children’s mental health problems or in paediatric care as an instrument to identify children with mental health problems, population‐specific norms and percentile cut‐off values are needed (Goodman *et al.*, 2012).

The Swedish version of the SDQ (SDQ‐Swe) has demonstrated adequate psychometric properties, with Chronbach’s alpha for the total problem scores at 0.76 and split‐half reliability of 0.78 (Smedje, Broman, Hetta & von Knorring, 1999), and has been validated for parental use in 5–15‐year‐old children (Malmberg, Rydell & Smedje, 2003) as well as self‐report for adolescents (Lundh, Wångby‐Lundh & Bjärehed, 2008). Recently, acceptable construct validity was concluded for parents’ and teachers’ ratings of Swedish preschool children (Dahlberg *et al.*, 2018). Concurrent validity of the teacher version of SDQ‐Swe has also been investigated, showing a moderate correlation (*r* = 0.65) between SDQ total problem scores and the teacher’s version of the CBCL for children aged 4–5 (Gustafsson, Gustafsson & Proczkowska‐Bjorklund, 2016). Smedje and colleagues (Smedje *et al.*, 1999) have derived parent‐reported norms for 6–10‐year‐old children, and Ghaderi, Kadesjö, Kadesjö and Enebrink (2014) have presented, but not published, parent‐reported data for Swedish 2–5‐year‐olds. The only peer‐reviewed and published data on children in Sweden younger than 6 years old come from Gustafsson, Proczkowska‐Björklund and Gustafsson (2017), who studied preschool teachers’ scores. Although the SDQ was not designed to be used with children under 2, the study by Gustafsson and colleagues included 1–5‐year old children. Furthermore, in their study, data were drawn from a relatively small sample considering the age span (*n* = 815) and 1 to 5‐year‐old children were grouped together, leaving age and gender specific differences not fully explored.

Previous research suggests that boys are scored as having more problems and less prosocial skills than girls (e.g. Davé, Nazareth, Senior & Sherr, 2008; Du, Kou & Coghill, 2008; Tobia, Gabriele & Marzocchi, 2013). Some studies report higher scores for girls on the emotional problems subscale, especially for school‐aged children (Capron, Thérond & Duyme, 2007; Mellor, 2005; Tobia *et al.*, 2013). Further, there are indications that younger children display higher scores on the total and hyperactivity/inattention scales but lower on the prosocial scale (Meltzer, Gatward, Goodman & Ford, 2003; Rothenberger, Becker, Erhart, Wille, Ravens‐Sieberer & Bella Study Group, 2008). The available normative data for young Swedish children (Ghaderi *et al.*, 2014) suggest that SDQ norms vary according to the age of the child in 1‐year age intervals. Age specific norms are also of great value when conducting studies, providing the researchers with the possibility of, for example, tracking changes over time or by assessing children at different ages with age appropriate cut‐off scores. For instance, a child could display changes in SDQ scores over time that are in line with normal development. Using the same cut‐offs across all ages would not take this development into account.

The above‐mentioned parent‐reported SDQ norms for 2–5‐year‐old children are available only in Swedish (Ghaderi *et al.*, 2014). The data were reported by age but generated based on relatively small subsamples wherein each age and gender subcategory were represented by approximately 200 children. Furthermore, data for teacher reported SDQ were lacking.

The use of the SDQ as a screening tool in clinical practice is increasing, and a multi‐informant and/or multiple context approach (providing assessments of the child from different informants and in different contexts, such as preschool and home) is considered to be the best practise when evaluating a child’s behavioral and emotional problems (Achenbach, McConaughy & Howell, 1987). Providing professionals in paediatric care with population‐specific norms and cut‐off values for both parent and teacher versions is therefore of importance for clinical decision‐making. Using information from multiple informants and contexts has also been emphasized for research purposes (Stone *et al.*, 2010).

Norms for instruments measuring behavior are often presented by gender, given known gender differences in mean scores. However, sometimes general norms can be more useful. Frick, Barry and Kamphaus (2005) argue that the different types of norms are useful for different purposes. For example, using gender‐specific norms would erase greater prevalence of any problems among girls or boys, which might not be desirable. Thus, we acknowledge the need to provide both general and gender‐specific norms and to identify how using the different cut off might impact the number of boys and girls who are identified as cases.

## Aim and hypotheses

The aim of the study at hand was to establish Swedish parent and preschool teacher SDQ norms for children aged 3–5, using data from a large community sample.

Based on previous studies, where significant age and gender differences in SDQ scores were found, we expected younger children to score higher than older children, and boys to score higher than girls on total scores and the related subscale scores. For the prosocial subscale, we expected the opposite differences. As shown in a previous study on Swedish preschool children (Fält, Wallby, Sarkadi, Salari & Fabian, 2018), we expected preschool teachers to report fewer problems compared with parents.

## Methods

### Data collection

Data were extracted from a population‐based intervention trial in Uppsala, Sweden, aiming at investigating the mental health of preschool children and their parents (reference has been removed to conceal the authors’ identities). All parents of children aged 3–5 were invited to fill in a set of questionnaires, including the SDQ, as part of their annual check‐up at child health centres. Questionnaires were sent home to each household, along with the invitation letter to the annual check‐up. Parents/guardians were asked to fill in one questionnaire each and bring the completed forms to the visit. In addition, parents were instructed to take a third questionnaire to the child’s preschool and ask the preschool teacher to complete the form, put it in the prepaid envelope provided and send it directly to the child health centre. In Sweden, more than 90% of all children aged 3–5 attend preschool (The Swedish National Agency for Education, 2013) and 95% visit the child health centres regularly (Wallby, Modin & Hjern, 2013). Thus, this study population was considered an adequate source of data from the general Swedish population of 3–5‐year‐olds.

The extracted data were mainly collected between August 2013 and August 2017. A study protocol, with detailed descriptions regarding the study design, field procedures and measures was published by Salari *et al.* (2013). The longitudinal design of the data collection means that a child could be present at one to three time‐points, since questionnaires could be filled in at 3, 4 and 5 years. Over four years, more than 28,000 sets of questionnaires were distributed in total; each set included three SDQs, two for parents and one for the preschool teacher. The average overall yearly consent rate in the study from which data were extracted was 39.1%.

### Sample

**Figure 1:**
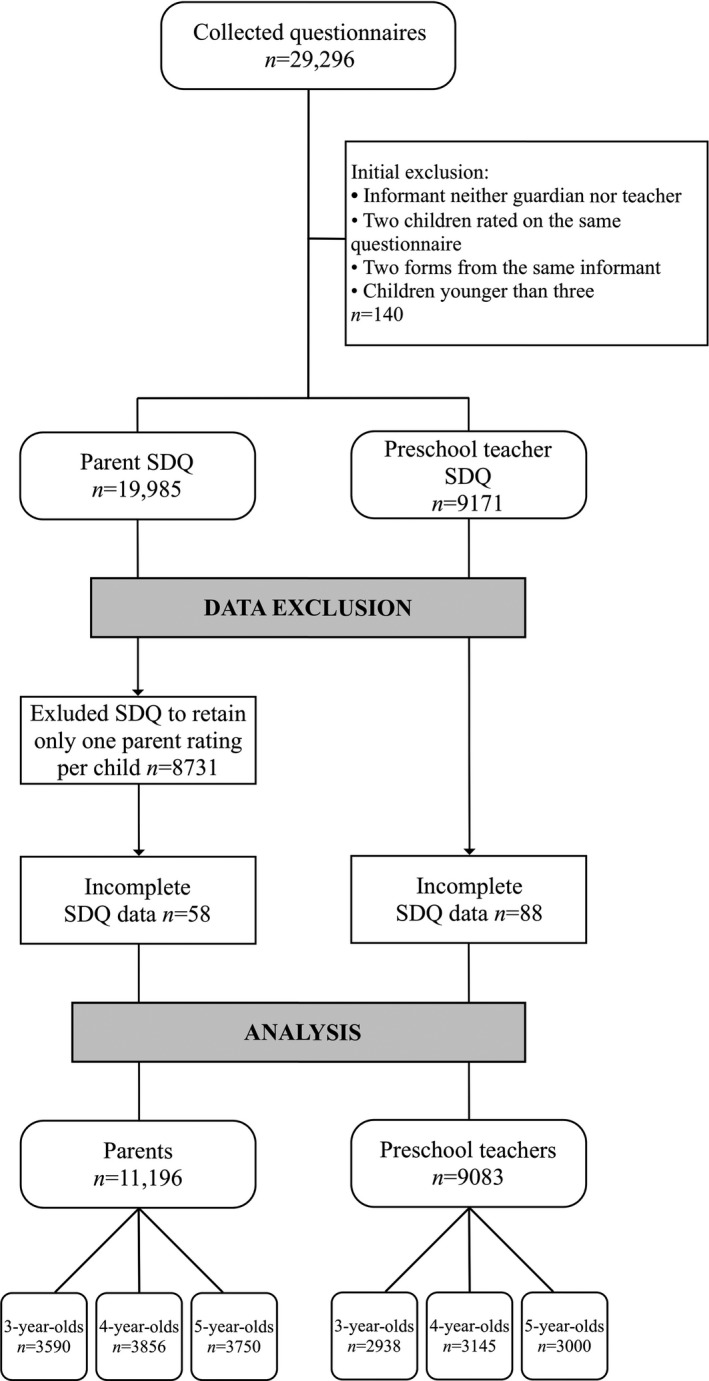
Flowchart of data exclusion.

A total of 29,296 questionnaires were collected from parents and teachers of pre‐schoolers aged 3–5. Data were excluded where informants were not the guardian or preschool teacher of the focal child, when more than one form was available from the same informant for the same child during the same year and when parents had rated two children on the same questionnaire. To assess subscale scores on the SDQ, at least three items per subscale need to be filled in (http://www.sdqinfo.com). Therefore, questionnaires with insufficient amount of data based on these restrictions were also excluded from statistical analyses. Since previous research on the same study population indicates a high inter‐rater agreement between mothers and fathers when rating preschool children (Fält, Wallby, Sarkadi, Salari & Fabian, 2017; Fält *et al.*, 2018), scores from parents were not analyzed separately for mothers and fathers. Instead, we generated “parent” SDQ variables consisting of either the mother’s or the father’s ratings. Where data for one child were available from both mothers and fathers, one questionnaire was selected at random. If more complete data were available from one parent, that parent was prioritized in the selection process. After data exclusion, 11,196 parent SDQ’s and 9,083 teacher SDQ’s, representing 12,245 children, remained for statistical analyses. Parents’ ratings constituted 6,239 mothers, 4,948 fathers, and 9 where both parents had completed the questionnaire together. The order of exclusion and number of excluded cases are displayed in Fig. 1.

The mean age of the parents was similar for all three child age groups, varying between 36.1 years and 38.2 years, with an overall age range between 19 and 72. Comparing the parental education level to sociodemographic data from the general Swedish population within the same age span, the sample distribution of parents’ education level was skewed towards higher education. Approximately 7/10 of the parents in the sample had studied beyond high school, while the corresponding number on a national level is 5/10 (http://www.scb.se). The proportion of parents born outside Sweden in the sample (~15%) was smaller than in the general population of Sweden (24%). Approximately nine out of ten children lived with both of their parents, which corresponds well to national data on preschool children (Statistics Sweden, 2013). See Table 1 for a breakdown of the sociodemographic variables of the sample.

**Table 1:** Sociodemographic characteristics of children and parents in the study sample

Sociodemographic variables	3‐year‐olds		4‐year‐olds		5‐year‐olds	
	*n*	%	*n*	%	*n*	%
Child gender						
Girl	1,769	49.3	1,908	49.5	1,813	48.3
Boy	1,815	50.6	1,946	50.5	1,934	51.6
Not specified	6	0.1	2	<0.1	3	0.1
Parent gender						
Female (mother)	2,034	56.7	2,170	56.3	2,035	54.3
Male (father)	1,553	43.3	1,684	43.7	1,711	45.6
Completed together	3	0.1	2	< 0.1	4	0.1
Parental highest level of education^a^						
High school or less	1,075	30.5	1,090	28.6	1,001	27.2
University	2,453	69.5	2,715	71.4	2,673	72.8
Parent country of birth^b^						
Sweden	3,008	84.2	3,229	84.6	3,128	84.8
Other	542	15.8	587	15.4	560	15.2
Parent relationship status^c^						
Single parent	150	4.3	210	5.5	253	6.9
Cohabiting	1,275	36.1	1,215	32.1	1,031	28.1
Married	2,052	58.2	2,298	60.6	2,331	63.5
Live‐apart	43	1.2	53	1.4	44	1.2
Other	7	0.2	13	0.3	12	0.3
Parent age (*M*, *SD*)^d^	36.1	5.4	37.1	5.3	38.2	5.4

a Missing data from 62 3‐year‐olds, 51 4‐year‐olds, and 76 5‐year‐olds.

b Missing data from 40 3‐year‐olds, 40 4‐year‐olds, and 62 5‐year‐olds.

c Missing data from 63 3‐year‐olds, 67 4‐year‐olds, and 79 5‐year‐olds.

d Missing data from 43 3‐year‐olds, 40 4‐year‐olds, and 69 5‐year‐olds.

### Instruments

The Swedish version of the SDQ was used to collect data from children 3–5 years old. The questionnaire is constituted of 25 items that make up five subscales measuring emotional symptoms, hyperactivity, conduct problems, peer problems, and prosocial behavior. Each subscale consists of five items scored on a three‐point Likert scale with 0 = not true, 1 = somewhat true and 2 = certainly true. Subscale scores range between 0 and 10, while the total difficulties score, generated by summing scores on all but the prosocial behavior scale, ranges between 0 and 40. Following discussions with health, research, and early education professionals involved in the *Children and Parents in Focus* study, some items were slightly modified for a clearer focus on behaviors rather than specific traits of individual children (see Table 2 for the SDQ items and modifications). The altered wording of the three items in question were considered to be in line with both pedagogical praxis in Sweden, and with the original intention of creating a questionnaire focusing on displayed behavior (Goodman, 1997). The construct validity and internal consistency of the SDQ with aforementioned alterations have been assessed with the *Children and Parents in Focus* study population in a previous study (Dahlberg *et al.*, 2018), concluding good internal consistency and support for the original SDQ structure with five subscales as proposed by Goodman (Dahlberg *et al.*, 2018; Goodman, 1997). Demographic information about the child (e.g. birthdate and gender) and the parent (e.g., education level, country of birth, relationship status) was collected together with the SDQ.

**Table 2:** The original five subscales of the SDQ. Modified items are presented in the footnotes

Subscale	Items
Conduct problems	Often has temper tantrums or hot tempers
	Generally obedient, usually does what adults request^a^
	Often fights with other children or bullies them
	Often argumentative with adults
	Can be spiteful to others^b^
Hyperactivity	Restless, overactive, cannot stay still for long
	Constantly fidgeting or squirming
	Easily distracted, concentration wanders
	Can stop and think things out before acting
	Sees tasks through to the end, good attention span
	Often complains of headaches, stomach‐aches or sickness
Emotional symptoms	Many worries, often seems worried
	Often unhappy, down‐hearted or tearful
	Nervous or clingy in new situations, easily loses confidence
	Many fears, easily scared
	Rather solitary, tends to play alone
Peer problems	Has at least one good friend
	Generally liked by other children
	Picked on or bullied by other children
	Gets on better with adults than with other children
	Considerate of other people's feelings
Prosocial behavior	Shares readily with other children (treats, toys, pencils, etc.)
	Helpful if someone is hurt, upset or feeling ill
	Kind to younger children^c^
	Often volunteers to help others (parents, teachers, other children)

a Usually does what adults request.

b Can behave spitefully towards others.

c Considerate of younger children.

### Statistical analysis

For this study, analyses were conducted in R version 3.5.2 (R Core Team, 2018) and SPSS 24 (IBM Corp., 2016). Due to the number of analyses and the large sample size, critical alpha was set to 0.001.

Parent and teacher SDQ subscale and total scores were assessed to identify borderline and abnormal bandings for the full sample as well as for girls and boys separately. Borderline cut‐offs were represented by the 80th percentile and abnormal cut‐offs by the 90th percentile (20th and 10th, respectively, for the prosocial scale). To investigate how using the different cut‐offs might impact the number of boys and girls who are identified as cases, we also calculated the percentage of girls and boys above the full sample and gender specific cut‐offs.

Means and standard deviations of the SDQ scores were calculated for the full sample as well as for girls and boys separately. Child gender differences in mean scores were assessed, along with effect sizes (partial eta squared) for all significant differences, using univariate ANOVA. To assess differences in SDQ total scores between preschool teachers and parents, separate independent *t*‐tests were performed for all three age groups.

Since the composition of our sample differed from the general population, we conducted linear regression analyses for each age group to assess the relation between SDQ total scores and child gender, parental education level, parent gender, and parents’ country of birth. Using linear regression enabled us to investigate the independent effect of each of these variables in a single analysis. Analyses were conducted separately for 3‐, 4‐, and 5‐year‐olds. The procedure was repeated for teacher scores, with parent gender omitted from the regression model.

## Results

Table 3 presents the suggested cut‐off scores for the SDQ‐Swe from the full sample. Table 4 contains gender‐specific cut‐offs. Preschool teachers reported significantly lower SDQ total scores than parents across child age groups (*t*_6180.3_ = 24.86, *p* < 0.001 for 3‐year‐olds; *t*_6698.3_ = 24.64, *p* < 0.001 for 4‐year‐olds; *t*_6527.6_ = 23.10, *p* < 0.001 for 5‐year‐olds). Ocular inspection of outcome in Tables 3 and 4 indicated that the cut‐offs for the preschool teachers’ ratings on SDQ total and subscales were generally lower than those of parents.

**Table 3:** Parent and teacher reported cut‐offs for the SDQ scales for the full sample, suggested by the 90th and 80th percentiles (10th and 20th for the prosocial scales)

	Parents		Preschool teachers	
	Borderline	Abnormal	Borderline	Abnormal
3‐year‐olds	*n* = 3,590		*n* = 2,938	
Total score	10	12	7	10
Emotional symptoms	2	3	1	2
Conduct problems	4	5	2	3
Hyperactivity/inattention	4	5	3	5
Peer problems	2	3	1	2
Prosocial	6	5	6	5
4‐year‐olds	*n* = 3,856		*n* = 3,145	
Total score	9	12	6	9
Emotional symptoms	2	3	1	2
Conduct problems	4	4	2	3
Hyperactivity/inattention	4	5	3	5
Peer problems	2	2	1	2
Prosocial	7	6	6	5
5‐year‐olds	*n* = 3,750		*n* = 3,000	
Total score	9	11	6	9
Emotional symptoms	2	3	1	2
Conduct problems	3	4	2	3
Hyperactivity/inattention	4	5	3	5
Peer problems	1	2	1	2
Prosocial	7	6	7	5

**Table 4:** Parent and teacher reported cut‐offs for the SDQ scales separate for girls and boys, suggested by the 90^th^ and 80^th^ percentiles (10^th^ and 20^th^ for the prosocial scales)

	Girls				Boys			
	Parents		Preschool teachers		Parents		Preschool teachers	
	Borderline	Abnormal	Borderline	Abnormal	Borderline	Abnormal	Borderline	Abnormal
3‐year‐olds	*n* = 1,769		*n* = 1,437		*n* = 1,815		*n* = 1,498	
Total score	10	12	6	9	11	13	8	11
Emotional symptoms	2	3	1	2	2	3	1	2
Conduct problems	4	4	2	3	4	5	2	4
Hyperactivity/inattention	4	5	3	5	4	5	4	5
Peer problems	2	2	1	2	2	3	1	3
Prosocial	7	6	6	5	6	5	5	5
4‐year‐olds	*n* = 1,908		*n* = 1,542		*n* = 1,946		*n* = 1,597	
Total score	9	11	5	8	10	13	7	11
Emotional symptoms	2	3	1	2	2	3	1	2
Conduct problems	3	4	1	2	4	4	2	4
Hyperactivity/inattention	3	5	2	4	4	5	4	6
Peer problems	1	2	1	2	2	3	1	2
Prosocial	7	6	7	6	6	5	6	5
5‐year‐olds	*n* = 1,813		*n* = 1,471		*n* = 1,934		*n* = 1,529	
Total score	8	10	4	7	9	12	7	10
Emotional symptoms	2	3	1	2	2	3	1	2
Conduct problems	3	4	1	2	3	4	2	3
Hyperactivity/inattention	3	4	2	4	4	5	4	5
Peer problems	1	2	1	2	2	2	1	2
Prosocial	8	6	8	7	7	6	6	5

The percentages of girls and boys above the 90th percentile were assessed using the SDQ total scores cut‐offs of parents and preschool teachers from the full sample as well as gender‐specific data (Table 5). The gender‐specific cut‐offs identified approximately 10–12% of all girls and boys as being above cut‐off across all ages, while the general cut‐offs had a wider range of identified cases (~7–16%) with boys being identified to a higher degree.

**Table 5:** Percentage of children scoring above the 90th percentile of the SDQ total score in the full sample (general cut‐offs) as well as in the samples of girls and boys (gender‐specific cut‐offs)

	Parents			Preschool teachers		
	Total *n*	General	Gender‐specific^a^	Total *n*	General	Gender‐specific^a^
3‐year‐olds						
Girls	1,769	11.2	11.2	1,437	7.8	10.9
Boys	1,815	15.6	11.6	1,498	13.6	10.8
4‐year‐olds						
Girls	1,908	8.4	11.0	1,542	8.1	10.4
Boys	1,946	13.4	10.9	1,597	15.8	11.1
5‐year‐olds						
Girls	1,813	9.4	12.1	1,471	6.5	10.9
Boys	1,934	14.9	11.9	1,529	13.9	11.2

a Theoretically, this amount should always be 10%. However, because the SDQ scores are discrete values, this number can be slightly above or below 10%.

Analysis of mean differences revealed significant mean differences between child genders on SDQ total scores and most subscales across ages and for both parents’ and preschool teachers’ ratings. The estimated effect sizes, however, ranged between insubstantial and small (please see Tables 6 and 7), using the commonly applied interpretations of effect size (Cohen, 1988; Lakens, 2013).

**Table 6:** Means and standard deviations of parent SDQ scores of girls and boys, with mean differences and significant effect sizes

	*M* (*SD*)			Mean difference 95% CI (boys‐girls)	Effect size (partial η^2^)
	Full sample	Girls	Boys		
3‐year‐olds					
Total score	6.8 (4.3)	6.4 (4.1)	7.1 (4.4)	0.4 to 1.0*	0.007
Emotional symptoms	1.1 (1.2)	1.1 (1.3)	1.1 (1.2)	−0.1 to 0.0	
Conduct problems	2.3 (1.7)	2.2 (1.7)	2.4 (1.7)	0.1 to 0.3	
Hyperactivity/inattention	2.4 (2.0)	2.2 (1.9)	2.6 (2.1)	0.3 to 0.5*	0.009
Peer problems	1.0 (1.3)	0.9 (1.2)	1.1 (1.4)	0.2 to 0.4*	0.010
Prosocial	7.9 (1.8)	8.2 (1.7)	7.6 (1.8)	−0.6 to −0.4*	0.022
4‐year‐olds					
Total score	6.2 (4.4)	5.7 (4.2)	6.7 (4.5)	0.7 to 1.3*	0.013
Emotional symptoms	1.2 (1.4)	1.1 (1.3)	1.2 (1.4)	0.0 to 0.2	
Conduct problems	2.0 (1.7)	1.9 (1.7)	2.1 (1.7)	0.0 to 0.2	
Hyperactivity/inattention	2.2 (2.0)	1.9 (1.9)	2.4 (2.1)	0.3 to 0.6*	0.014
Peer problems	0.8 (1.3)	0.7 (1.1)	0.9 (1.3)	0.2 to 0.3*	0.012
Prosocial	8.2 (1.7)	8.4 (1.6)	7.9 (1.8)	−0.6 to −0.4*	0.021
5‐year‐olds					
Total score	5.5 (4.3)	5.0 (3.9)	6.0 (4.6)	0,8 to 1.3*	0.015
Emotional symptoms	1.2 (1.4)	1.2 (1.4)	1.2 (1.4)	0.0 to 0.2	
Conduct problems	1.7 (1.6)	1.6 (1.6)	1.8 (1.7)	0.1 to 0.3*	0.005
Hyperactivity/inattention	1.9 (2.1)	1.6 (1.9)	2.2 (2.2)	0.4 to 0.7*	0.017
Peer problems	0.7 (1.2)	0.6 (1.0)	0.8 (1.2)	0.1 to 0.3*	0.008
Prosocial	8.5 (1.6)	8.7 (1.5)	8.2 (1.7)	−0.6 to −0.4*	0.024

* *p* < 0.001

**Table 7:** *Means and standard deviations of preschool teacher SDQ scores of girls and boys, with mean differences and significant effect* sizes

	*M* (*SD*)			Mean difference 95% CI (boys‐girls)	Effect size (partial η^2^)
	Full sample	Girls	Boys		
3‐year‐olds					
Total score	4.1 (4.4)	3.6 (3.8)	4.6 (4.9)	0.7 to 1.3*	0.013
Emotional symptoms	0.6 (1.1)	0.6 (1.0)	0.6 (1.1)	−0.1 to 0.1	
Conduct problems	1.1 (1.6)	0.9 (1.4)	1.2 (1.7)	0.1 to 0.3*	0.006
Hyperactivity/inattention	1.8 (2.2)	1.5 (1.9)	2.0 (2.4)	0.4 to 0.7*	0.014
Peer problems	0.7 (1.3)	0.6 (1.1)	0.8 (1.4)	0.1 to 0.3*	0.008
Prosocial	7.9 (2.2)	8.3 (2.0)	7.6 (2.3)	−1.0 to 0.4*	0.024
4‐year‐olds					
Total score	3.6 (4.4)	2.8 (3.6)	4.4 (4.9)	1.2 to 1.8*	0.030
Emotional symptoms	0.5 (1.1)	0.5 (1.1)	0.6 (1.1)	0.0 to 0.1	
Conduct problems	0.9 (1.6)	0.7 (1.2)	1.2 (1.8)	0.3 to 0.5*	0.019
Hyperactivity/inattention	1.6 (2.3)	1.2 (1.9)	2.1 (2.5)	0.7 to 1.0*	0.036
Peer problems	0.5 (1.1)	0.4 (1.0)	0.6 (1.2)	0.1 to 0.2*	0.006
Prosocial	8.3 (2.1)	8.6 (1.8)	7.9 (2.3)	−1.0 to −0.5*	0.034
5‐year‐olds					
Total score	3.1 (4.1)	2.4 (3.5)	3.8 (4.5)	1.1 to 1.7*	0.029
Emotional symptoms	0.5 (1.0)	0.5 (1.0)	0.5 (1.1)	−0.1 to 0.1	
Conduct problems	0.8 (1.5)	0.6 (1.2)	1.0 (1.7)	0.3 to 0.5*	0.021
Hyperactivity/inattention	1.4 (2.1)	0.9 (1.8)	1.8 (2.3)	0.7 to 1.0*	0.043
Peer problems	0.4 (1.0)	0.4 (0.9)	0.5 (1.0)	0.0 to 0.2	
Prosocial	8.6 (2.0)	9.0 (1.6)	8.2 (2.2)	−1.1 to − 0.6*	0.047

* *p* < 0.001.

The regression models revealed significant associations between ratings of total SDQ scores and most background variables across child age (Tables 8 and 9). As expected, male gender of the child was significantly associated with more behavior problems. Low parental education was associated with more problems, as were ratings from parents born outside Sweden. Fathers’ ratings were associated with higher parent SDQ scores for 3‐ and 4‐, but not 5‐year‐olds.

**Table 8:** Beta‐coefficients (95% CI) from linear regression analyses of background variables and SDQ total scores rated by parents

	Dependent variable		
	SDQ Total score		
	3‐year‐olds (*n* = 3,516)	4‐year‐olds (*n* = 3,796)	5‐year‐olds (*n* = 3,666)
Child gender (girl)	0.732* (0.455, 1.008)	0.988* (0.715, 1.261)	1.031* (0.757, 1.305)
Parental education level (low)	−0.739* (−1.041, −0.437)	−1.073* (−1.376, −0.771)	−0.885* (−1.193, −0.576)
Parent gender (mother)	0.490* (0.606, 1.382)	0.486* (0.210, 0.761)	0.097 (−0.179, 0.372)
Parent country of birth (Sweden)	0.994* (0.606, 1.382)	0.873* (0.494, 1.252)	1.141* (0.759, 1.523)

The reference category for each variable is stated in brackets.

* *p* < 0.001.

**Table 9:** Beta‐coefficients (95% CI) from linear regression analyses of background variables and SDQ total scores rated by preschool teachers

	Dependent variable		
	SDQ Total score		
	3‐year‐olds (*n* = 2,588)	4‐year‐olds (*n* = 2,775)	5‐year‐olds (*n* = 2,629)
Child gender (girl)	0.992* (0.666, 1.318)	1.431* (1.117, 1.744)	1.419* (1.121, 1.718)
Parental education level (low)	−0.565 (−0.922, −0.208)	−0.706* (−1.055, −0.358)	−0.666* (−1.008, −0.324)
Parent country of birth (Sweden)	1.187* (0.722, 1.651)	0.494 (0.045, 0.943)	0.325 (−0.103, 0.752)

The reference category for each variable is stated in brackets.

* *p* < 0.001.

## Discussion

In the study at hand, we set out to establish separate parent and preschool teacher SDQ norms for Swedish children aged 3–5. We also examined gender differences and the impact of background variables on SDQ scores.

Given our study’s implication that preschool children scored lower with increased age, we argue for the use of age‐specific cut‐offs for preschool children. This is also in line with previous research. However, in our sample, a child could be rated at one, two, or three time‐points, making direct statistical comparisons between age groups difficult due to clustering effects.

As can be seen from Tables 4 and 5, for a few subscales and for some ages, the abnormal and borderline cut‐offs were identical. This could be due to the rather narrow range of the SDQ subscales and the cut‐offs being integer values without fractional components. There might also be a floor effect, suggested by the rather low average scores, which could affect the variability of the scores. This, however, was not gauged statistically in this study, but may be of interest for future studies to investigate further.

On average, boys scored higher than girls in our study, which is in line with most international SDQ studies. A question that arose when planning this study was whether gender‐specific cut‐offs should be presented or not. Looking at the available literature on the SDQ, practises differ greatly and no clear instructions are provided from Goodman’s studies or the sdqinfo.com website. In our study, using the same cut‐offs for boys and girls resulted in more boys being categorized as having abnormal behavior problems, which is in line with research on the prevalence of psychiatric disorders related to behavior problems (Costello, Mustillo, Erkanli, Keeler & Angold, 2003). Analyzing the mean differences between genders, the effect sizes were found to be small, which implies that the emotional and behavioral problems measured through the SDQ are complex and probably influenced by many factors, including gender. One argument for the use of gender‐specific norms is that this takes the systematic difference between genders into account and does not jeopardize the normal distribution. However, such potential gender differences could also be assessed through analyzing mean differences in scores. On the other hand, using the same cut‐offs for both genders provides us with the ability of easily capturing girls and boys above the normal range of SDQ scores. Since gender differences merely explained a small amount of variance in scores in our sample, using separate norms for girls and boys might not be of value from a clinician’s point of view, but could complicate scoring procedures. From an epidemiological stance, however, the differences could be of great importance. Depending on viewpoint, the question of whether to use gender‐specific cut‐offs or not can thus be answered differently. Therefore, we chose to provide the reader with both combined norms and separately for girls and boys.

Internationally, the SDQ cut‐offs are higher than or similar to the ones provided in the present study (Borg *et al.*, 2014; Mellor, 2005; Tobia & Marzocchi, 2018). Comparing our cut‐offs with previous Swedish norms, results harmonize well for parents’ ratings (Ghaderi *et al.*, 2014), with minor differences on subscale level for some age groups. However, preschool teachers’ cut‐offs were lower in our study than previously published data (Gustafsson *et al.*, 2017), where they are more similar to this study’s parent ratings. One possible explanation of this difference could be that the previous norms were combined for 1–5‐year‐olds.

Parents with lower education reported more problems, indicated by the results from the regression analyses. Lower parental education levels and lower household income have been associated with negative and coercive parenting practices and externalized behavior problems in children (Bøe, Sivertsen, Heiervang, Goodman, Lundervold & Hysing, 2014; Strohschein, 2005), which might explain the differences between ratings from high and low educated parents to some extent. The higher ratings could also be due to inadequate expectations on children’s behavior or more negative parental perceptions due to generally more stressful circumstances, regarding housing, economy or work demands.

Preschool teachers rated the children lower than parents, which is contrary to previous findings from other Nordic countries, such as Denmark and Finland, where scores were quite similar for parents and teachers (Borg *et al.*, 2014; Elberling, Linneberg, Olsen, Goodman & Skovgaard, 2010; Niclasen *et al.*, 2012). However, there are also studies suggesting that teachers report lower levels of behavior and emotional problems compared to parents (Verhulst & Akkerhuis, 1989; Winsler & Wallace, 2002). Sweden differs from many European and non‐European countries in that the preschool institution is characterized by a philosophy of child‐centered care, quite different from the more normative approach of schools (Broström, 2006; The Swedish National Agency for Education, 2018). Thus, children might either display less emotional and behavioral problems in the preschool setting, or preschool teachers might perceive any such problems as signifying a problem not within the child, but within the preschool environment, thus lowering the proportion of behaviors reported as problematic. In addition, results in a previous study on preschool teachers’ experiences of using the SDQ in the Swedish preschool setting (Fält, Sarkadi & Fabian, 2017) show that the use of structured assessment forms is considered to be contradictory to the preschool philosophy. Findings from the same study also indicate that teachers are worried about parents’ reactions and fear both making incorrect assessments and labelling children. Teachers underreporting children’s problems might thus be another possible explanation for the lower SDQ scores reported by teachers. However, it should be noted that although the level of reported SDQ scores differed between teachers and parents, the patterns of the cut‐off values (decreasing with the age of the children) were rather similar for both informants. This finding implies that the teachers’ ratings are based on sincere assessments of the children’s behavior, and does not reflect falsely low ratings.

## Conclusion

The current study uses data from a large community sample to provide norms for children aged 3–5 years, based 29,296 SDQ forms collected from mothers, fathers, and preschool teachers. Preschool teachers’ scores were generally lower than parents’, which contradicts some previous studies. Differences between girls and boys were found, suggesting that boys display higher levels of behavior problems. Parental education and country of birth affected scores, where lower parental education and country of origin outside of Sweden were associated with more difficulties. In the current study, cut‐offs for total scores and all the subscales are presented for each age group, gender and rater category. General norms, based on cut‐offs without gender separation, are also provided for each age group and for both informant groups. This study adds to the previous knowledge and research on the SDQ in that it provides norms for both parents and preschool teachers for 3–5‐year‐olds. Population‐specific norms and percentile cut‐offs provided in this study facilitate identifying children in need of interventions in paediatric care and enable cross‐country comparisons of children’s mental health problems.

## Conflict of interest

The authors declare that they have no conflict of interest.
